# Quantification of Daily-Living Gait Quantity and Quality Using a Wrist-Worn Accelerometer in Huntington's Disease

**DOI:** 10.3389/fneur.2021.719442

**Published:** 2021-10-27

**Authors:** Karin Keren, Monica Busse, Nora E. Fritz, Lisa M. Muratori, Eran Gazit, Inbar Hillel, Micky Scheinowitz, Tanya Gurevich, Noit Inbar, Nurit Omer, Jeffrey M. Hausdorff, Lori Quinn

**Affiliations:** ^1^Center for the Study of Movement, Cognition and Mobility, Neurological Institute, Tel Aviv Sourasky Medical Center, Tel Aviv, Israel; ^2^Department of Biomedical Engineering, Tel Aviv University, Tel Aviv, Israel; ^3^Centre for Trials Research, Cardiff University, Cardiff, United Kingdom; ^4^Departments of Health Care Sciences and Neurology, Wayne State University, Detroit, MI, United States; ^5^Department of Physical Therapy, School of Health Technology and Management, Stony Brook University, Stony Brook, NY, United States; ^6^George Huntington's Institute, Muenster, Germany; ^7^School of Public Health, Tel Aviv University, Tel Aviv, Israel; ^8^Movement Disorders Unit, Tel Aviv Medical Center, Tel Aviv, Israel; ^9^Sackler School of Medicine and Sagol, School of Neuroscience, Tel Aviv University, Tel Aviv, Israel; ^10^Department of Physical Therapy, Sackler Faculty of Medicine, Tel Aviv University, Tel Aviv, Israel; ^11^Rush Alzheimer's Disease Center and Department of Orthopaedic Surgery, Rush University Medical Center, Chicago, IL, United States; ^12^Department of Biobehavioral Sciences, Teachers College, Columbia University, New York, NY, United States

**Keywords:** accelerometry, physical activity, digital markers, inertial measurements, chorea

## Abstract

**Background:** Huntington's disease (HD) leads to altered gait patterns and reduced daily-living physical activity. Accurate measurement of daily-living walking that takes into account involuntary movements (e.g. chorea) is needed.

**Objective:** To evaluate daily-living gait quantity and quality in HD, taking into account irregular movements.

**Methods:** Forty-two individuals with HD and fourteen age-matched non-HD peers completed clinic-based assessments and a standardized laboratory-based circuit of functional activities, wearing inertial measurement units on the wrists, legs, and trunk. These activities were used to train and test an algorithm for the automated detection of walking. Subsequently, 29 HD participants and 22 age-matched non-HD peers wore a tri-axial accelerometer on their non-dominant wrist for 7 days. Measures included gait quantity (e.g., steps per day), gait quality (e.g., regularity) metrics, and percentage of walking bouts with irregular movements.

**Results:** Measures of daily-living gait quantity including step counts, walking time and bouts per day were similar in HD participants and non-HD peers (*p* > 0.05). HD participants with higher clinician-rated upper body chorea had a greater percentage of walking bouts with irregular movements compared to those with lower chorea (*p* = 0.060) and non-HD peers (*p* < 0.001). Even after accounting for irregular movements, within-bout walking consistency was lower in HD participants compared to non-HD peers (*p* < 0.001), while across-bout variability of these measures was higher (*p* < 0.001). Many of the daily-living measures were associated with disease-specific measures of motor function.

**Conclusions:** Results suggest that a wrist-worn accelerometer can be used to evaluate the quantity and quality of daily-living gait in people with HD, while accounting for the influence of irregular (choreic-like) movements, and that gait features related to within- and across-bout consistency markedly differ in individuals with HD and non-HD peers.

## Introduction

Huntington's disease (HD) is an inherited neurodegenerative disease that results in impairments in motor, cognitive, and behavioral domains (1). Exciting, novel therapeutics are currently under development for treating HD (2). One key to demonstrating the utility of these emerging therapeutics is to use clinical endpoints that reflect real-life impact. Quantitative, objective measurement of gait and physical activity in daily-living, ambient settings have the potential to be highly informative; however, the presence of involuntary movements (e.g., chorea) in people with HD poses a challenge for accurate assessment.

There is increasing interest in wearable, remote monitoring sensors to evaluate walking and daily-living physical activity with minimal operator input in a range of neurological disorders including Parkinson's disease (3–5), multiple sclerosis (6–8), and HD (9–12). Several studies have begun to evaluate the challenges associated with activity monitoring in HD. Inertial measurement units (IMUs) are able to accurately estimate spatiotemporal gait measures in people with HD in an in-lab, supervised setting (13). However, to date, IMUs are not able to be easily employed in home and daily living environments. Accelerometer-based assessments using wrist or trunk-worn devices have been used in several studies in people with HD (10, 14). Dinesh et al. (10) used accelerometer-based assessments for clinic and home-based assessments of people with HD, and reported differences in walking amounts, speed and time spent lying down compared to non-HD peers. However, no study to date has specifically compared in clinic vs. at home accelerometry-based assessments. Furthermore, the possible influence of involuntary or irregular movements (12), which are common in people with HD, on walking and activity counts have not been evaluated.

Choreic movements are a hallmark symptom of HD and are often the first motor symptom, affecting almost any part of the body (1). While several studies have used wearable devices to quantify arm chorea in static positions (10, 15, 16), no studies have specifically considered the influence of chorea and other involuntary or irregular movements on walking and daily-living physical activity. Typical walking has a consistent pattern of rhythmicity, and irregular movements, which can be defined as movements that deviate from the typical rhythmic walking pattern, may influence the signal processing methods that are typically used to extract measures of activity and gait (for example, from wrist-worn devices). Methods that account for the presence of such involuntary movements during the performance of complex tasks such as walking are needed to assure that measures of gait and daily-living physical activity reflect the walking pattern and are not just a reflection of the irregular movements.

As a step forward towards the use of wearable, 24/7 measurement of daily-living physical activity and mobility in HD, we aimed to evaluate everyday walking and activity taking into account irregular (e.g., chorea-like) movements. We specifically addressed the following questions: (1) What percent of walking during the day is spent with irregular (upper-extremity) movements in HD and age-matched non-HD peers? (2) Is daily-living gait quantity and quality different among individuals with HD compared to non-HD peers? and (3) How are measures of daily-living walking quantity and quality related to HD disease-specific measures including clinician ratings of motor function, chorea, and dystonia?

## Methods

### Participants

HD participants (*n* = 42) and age-matched non-HD peers (*n* = 22) from clinical centers in four countries were recruited for this study. Inclusion criteria for HD participants were: (1) aged 18 years or older; (2) genetically confirmed HD; (3) Total Functional Capacity (TFC)≥7; and (4) able to walk 10 meters independently without assistive device. Exclusion criteria were: (1) diagnosis of juvenile-onset HD; (2) history of co-morbid neurological conditions such as stroke or multiple sclerosis; and (3) acute orthopedic conditions (e.g., ankle sprain, fracture). Inclusion criteria for non-HD peers were: (1) aged 18 years or older; and (2) able to walk 10 meters independently without assistive device. Exclusion criteria were: (1) history of neurological conditions such as stroke or multiple sclerosis; and (2) acute orthopedic conditions (e.g., ankle sprain or fracture).

### Demographic and Clinical Profile

Participants attended one in-clinic assessment lasting approximately 90 min. Demographic data (e.g., age, gender, height, weight) was collected and, for the HD participants, disease-specific assessments were completed or were obtained from clinical records. Disease-specific status was quantified through the Unified Huntington's Disease Rating Scale (UHDRS) (17) including the TFC, Total Motor Score (TMS), Functional Assessment (FA), Independence Scale, and the Symbol Digit Modality Test (SDMT). TMS scores were administered by certified raters at each site.

### In Clinic Assessment

HD participants (*n* = 42) and age-matched non-HD peers (*n* = 14) completed the in clinic assessment. We used a research-grade inertial measurement unit with a tri-axial accelerometer, magnetometer and gyroscope (Opal inertial sensors (APDM, Inc) Activinsights; 43.7 mm × 39.7mm × 13.7 mm; weight: < 25 grams; 128 Hz sampling rate) to quantify movement parameters during the performance of a standardized laboratory-based circuit of functional activities, required for independent activities of daily living in the home. This included quiet sitting for 30 sec, writing a sentence, drinking a 100 ml glass of water, 5-repetition chair stand test, Timed Up & Go (TUG) Test, 2-min walk test, step-ups, and quiet standing with eyes open and closed. These activities were used to train and test an algorithm for the automated detection of walking. All activities were videorecorded using a GoPro camera. The algorithm is described in the Supplementary Material.

### Everyday Walking Assessment

#### 7-Day Monitoring

At the end of the in-clinic testing session, 29 HD participants and 22 age-matched non-HD peers were given a research-grade tri-axial accelerometer (GENEActiv, Activinsights; 43 × 40 × 13 mm; weight: 16 g; 100 Hz sampling rate) to wear on their wrist for 24 h over the next 7 days. To minimize the impact of everyday movements like hand-writing, teeth-brushing and gestures that are typically conducted with the dominant hand, the accelerometer was placed on the wrist of the non-dominant hand and participants were asked to continue their activities as usual. Upon completion of the 7-day period, participants removed the device and sent it back to the local clinical site, using a provided addressed, stamped and padded envelope.

Using the automated algorithm that is described below in section Automatic Identification of Walking Bouts and in further detail in the Supplementary Material, each bout (i.e., window) of walking throughout the 7-day recording was identified. To minimize the possible impact of “wear time”, we only included participants with at least 3 full day recordings (18) with at least 10 hours of wear time within a 12 hour range (8:00 AM to 8:00 PM). Five participants from the HD group and one participant from the non-HD peer group who did not meet these conditions were excluded from the gait quantity analysis. Previously reported measures that reflect the quantity and quality of walking were then extracted, as detailed elsewhere (8, 18–20). We used bouts that were at least 6 sec to be able to recognize a periodic movement from a wrist-worn signal to evaluate the amount of walking (21).

#### Automatic Identification of Walking Bouts

The first stage in the automatic detection of the walking bouts included low pass filtering and removal of the DC component of the accelerometer signals. The Euclidean norm of the 3D accelerometer signal was computed to minimize any effects of the placement of the sensor on the wrist and angle or orientation dependency. An activity threshold was set to 0.10 [g] to eliminate areas in the recording with no activity. The remainder of the areas that crossed the threshold was divided into windows with a duration of 6 sec, with 5-second overlap, for further processing. A second threshold based on a standard deviation of minimum a 0.10 [g] was applied to each window of the acceleration signal. Using power spectral density (PSD) as computed with Welch's method in each window, the frequency at the maximum power was calculated and examined. Windows with a peak frequency within a “gait” range 0.5–3 [Hz] were further examined to evaluate the consistency during the walking bout using autocorrelation. Windows that passed all the thresholds and conditions mentioned above were identified as windows that contain walking (see Supplementary Figure 1). The algorithm output was validated in two ways: (1) comparison to a previously validated algorithm that used IMU signals from sensors placed on the ankles to detect walking and, (2) comparison to a database with and without walking that were annotated using a GoPro camera (see Supplementary Material for further details).

#### Automatic Identification of Walking Bouts With Irregular Movements

The algorithm for detecting “irregular” walking was developed to detect walking bouts that likely contain irregular (chorea-like) wrist movements. The detection was based on four features from the recorded accelerometer signals using empirically derived thresholds that were based on 2-min walk trials that were performed in laboratory settings in non-HD peers and people with HD with varying degrees of upper extremity chorea. Based on video recordings of these walks, segments with no chorea, mild chorea, and severe upper extremity chorea were identified to determine the threshold for defining the presence or absence of irregular (chorea-like) walking bouts (see Supplementary Material for further details).

#### Gait Quality

To evaluate the quality of daily-living walking, we focused on walking bouts that were at least 30 sec long, as these likely reflect steady-state walking (8, 19, 22, 23). To ensure that these measures were reflective of everyday walking, at least ten walking bouts were required for each participant to be included in the analyses. Because of these constraints, one participant from the HD group and from the non-HD peer group was excluded from the quality analysis.

The percentage of walking bouts with irregular upper extremity movements was quantified and, subsequently, walking bouts that had movements were removed before determining gait quality (see Supplementary Material). The number of walking bouts with irregular movements was divided by the total number of walking bouts to determine the percentage of walking bouts that contain irregular movements for each participant. To investigate the association between the amount of detected irregular walking bouts and clinical ratings of HD symptoms, the results were divided into 3 groups: non-HD peers, HD with high upper body chorea and HD with low upper body chorea (defined by average TMS ratings for right and left arm) and confirmed by video review. An average score <2 was defined as low chorea and ≥2 was defined as high chorea.

#### Gait Quantity

The amount of walking was calculated by summing the time spent walking and the steps of all walking bouts of every day during the week, from windows equal or longer than 6 sec. For each participant, the median (across the week) daily walk time and step counts were calculated. Total daily-living physical activity was defined by calculating the signal vector magnitude (SVM) of the measured acceleration signal from three axes of each 60 second window during the day (24). For each day, the mean of all windows was calculated, and the median value of all days was extracted for each participant. This parameter represented total daily-living physical activity.

Several domains of gait quality were evaluated, adapting those proposed previously when other sensor locations were used (8, 18–20). To evaluate the walking rhythm, the cadence and dominant frequency of the acceleration signal in the frequency domain were extracted. The magnitude of the signal, a measure that reflects the amount of movement within each walking bout, was measured by quantifying the range and root-mean-square (RMS) of the acceleration signal's squared vector magnitude of each walking bout (23, 25, 26). To evaluate the within-bout walking consistency, we extracted the step and stride regularity and the amplitude and width peak of the dominant frequency at the power spectral density (PSD) spectrum in each bout. Step and stride regularity are measures of within-bout walking consistency of the step and stride patterns; values can range from 0 to 1, with higher values indicating greater consistency of the step-to-step and stride-to-stride temporal patterns, respectively (25). The width is a measure of the frequency dispersion and is related to variability (in a sense, the inverse of the consistency). The amplitude of the peak in the frequency domain reflects the dominance (or strength) of the frequency in the signal (8, 23, 25, 27). In addition, to assess bout-to bout variations over the week, i.e., how variable a participant's walking pattern is across the many walking bouts of the week, we calculated the standard deviation (SD) of each of the walking quality measures for each participant (20).

### Statistical Analysis

The normality of the data was tested with a Shapiro-Wilk test. Descriptive statistics are reported as mean ± SD for data that were distributed normally and median (inter-quartile range, IQR), for non-normal distributions. Spearman's correlations assessed the relationship between daily-living walking measures and HD clinical scores. To compare groups, Mann-Whitney tests were used for non-normal distributions and independent *t*-tests were used for normal distribution. For the within-bout and across-bout walking gait quality measures, we used a Bonferroni correction to define the level of significance (alpha = 0.01); otherwise, a two-tailed *p-*value < 0.05 was considered statistically significant. Effect sizes were calculated by dividing the test statistic (Z) by the squared number of observations (N) for results that were not distributed normally and Cohen's d for normal distributions (28). Statistical analyses were performed using SPSS Version 27 and Python (libraries: Pandas, Numpy, pingouin, Scipy, Matplotlib).

## Results

HD participants and non-HD peers were similar with respect to age, gender, height, and weight (Table 1). Supplementary Table 3 summarizes the in-lab gait of the two groups as measured during the 2 min walk. Based on measurements from the sensors placed on the legs, gait speed, one measure of magnitude and one measure of consistency were lower in people with HD compared to non-HD peers. Other gait quality measures did not differ in the two groups (*p* > 0.05) (see Supplementary Material for further details).

**Table 1:** Participant characteristics.

	**HD participants**	**Non-HD peers**	***P*-value**
*n*	29	22	-
**Demographics**			
Age (yrs)	56.55 ± 11.90	53.00 ± 9.64	0.265
Gender (% women)	49%	56%	0.492^†^
Weight (kg)	72.85 ± 14.35	79.43 ± 14.25	0.110
Height (cm)	173.42 ± 9.06	170.8 ± 10.40	0.324
**HD clinical rating scores**			
UHDRS Total Motor Score (TMS)	42.90 ± 17.00	-	-
Total Functional Capacity (TFC)	11 ± 2	-	-
Functional Assessment	21 ± 2	-	-
SDMT	20 ± 13	58 ± 17^*^	<0.001

*Values are mean+/-SD, except as indicated for gender*.

† *Chi square test. SDMT, symbol digit modality test; UHDRS, Unified Huntington Disease Rating Scale*;

* *SDMT for non-HD peers calculated from 10 participants only*.

Table 2 summarizes the quantity and quality of daily-living gait in the two groups. Although participants with HD spent less time walking during the day and had lower step counts compared to non-HD peers, these differences were not significant (*p* = 0.33 and *p* = 0.13 respectively). The number of all walking bouts (or windows) per day also did not differ in the two groups (*p* = 0.24); however, there was a trend for participants with HD to have fewer long walking bouts (>30 sec) during daily-living (*p* = 0.053). The total daily-living physical activity was significantly greater for non-HD peers compared to HD participants (*p* = 0.006).

**Table 2:** Daily-living gait quantity and quality measures in HD and non-HD peers.

**Daily-living gait quantity**		**HD participants**	**Non-HD peers**	***P*-value**	**Effect size**
		**(*n =* 24)**	**(*n =* 21)**		
Walking time per day (min)		73.23 ± 31.81	80.94 ± 18.32	0.33	0.29
Steps per day		7458 ± 3368	8758 ± 2041	0.13	0.46
Total number of walking bouts per day		244.125 ± 87.42	216.59 ± 65.29	0.24	0.35
Number of long walking bouts (≥30 sec) per day		22.92 ± 13.47	30.05 ±10.03	0.053	0.59
Total daily-living activity index (mg)		42.13 (0.41)	42.93 (4.22)	0.006^*^ ^†^	0.39
**Daily-living gait quality**		**HD participants**	**Non-HD peers**	***P*** **-value**	**Effect size**
		**(*****n =*** **28)**	**(*****n =*** **21)**		
Rhythm	Cadence (step/min)	105.02 ± 8.98	109.76 ± 5.35	0.04	0.62
	Dominant frequency (Hz)	1.81 ± 0.18	1.86 ± 0.12	0.25	0.34
Magnitude	Range (g)	1.74 ± 0.35	1.66 ± 0.24	0.42	0.24
	RMS (g)	0.26 ± 0.05	0.30 ± 0.06	0.015^*^	0.73
Regularity/ Consistency	Amplitude dominant frequency (unitless)	0.67 ± 0.15	0.87 ± 0.14	<0.001^*^	1.45
	Width dominant frequency (Hz)	0.83 (0.21)	0.70 (0.02)	<0.001^*^	0.72
	Step regularity (unitless)	0.40 ± 0.15	0.66 ± 0.07	<0.001^*^	2.08
	Stride regularity (unitless)	0.33 (0.24)	0.70 (0.08)	<0.001^*^	0.72
	Step time variability (%)	26.27 ± 5.23	18.63 ± 6.62	<0.001^*^	1.30

* *Significant after Bonferroni correction (p = α/components; α= 0.05)*.

† *The total daily-living physical activity index looks similar in both groups, however, statistical analysis reveals a small but significant group difference. Gait Quantity: Entries are reported as mean+/-SD or median (IQR) determined on each participant across all walking bouts that were equal or larger than 30 sec. Data are presented based on walking bouts equal to or longer than 6 sec. Values are presented as mean+/-SD or median (IQR), before removing irregular bouts (results after removing irregular bouts can be found in Supplementary Material); Gait Quality: Values are presented as mean+/-SD or median (IQR) determined for all walking bouts that were equal or larger than 30 sec, after removing irregular bouts*.

**Figure 1:**
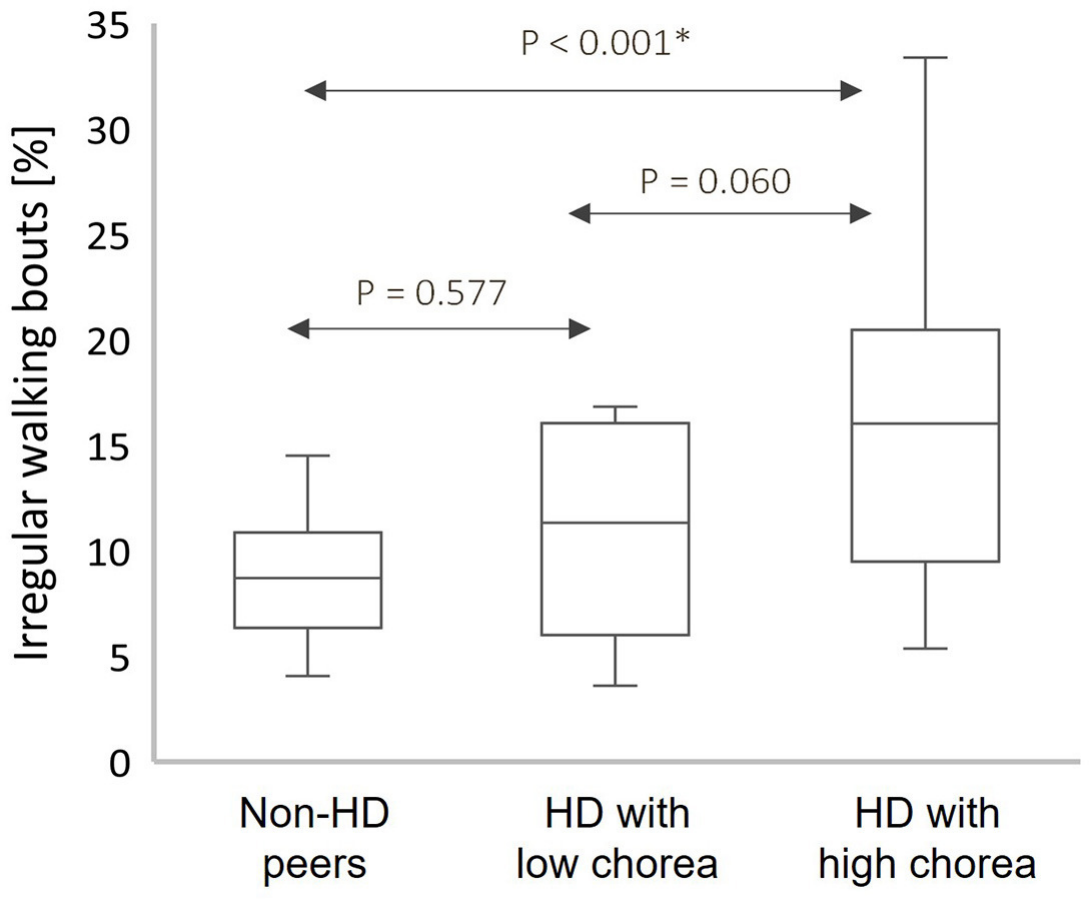
Box plots of the percentage of irregular walking bouts (WB) from the total count of WB among the HD participants and non-HD peers with high upper body chorea score (High) and with low upper body chorea score (Low); box plots present the median, interquartile range (IQR), upper limit (third quartile +1.5*IQR), and lower limit (first quartile −1.5*IQR); The percentage of walking bouts identified as associated with irregular movements was greater in participants with greater levels of observer-rated upper body chorea. *Significant after Bonferroni correction for multiple comparisons.

Figure 1 shows the percentage of walking bouts that were identified as having irregular movements for all participants. Among the HD participants with high average upper body chorea scores (≥2), the percentage of irregular walking bouts was greater than that seen among the HD participants with low clinical scores of chorea (*p* = 0.060) and almost twice as large as that seen in the non-HD peers (*p* < 0.001).

**Figure 2:**
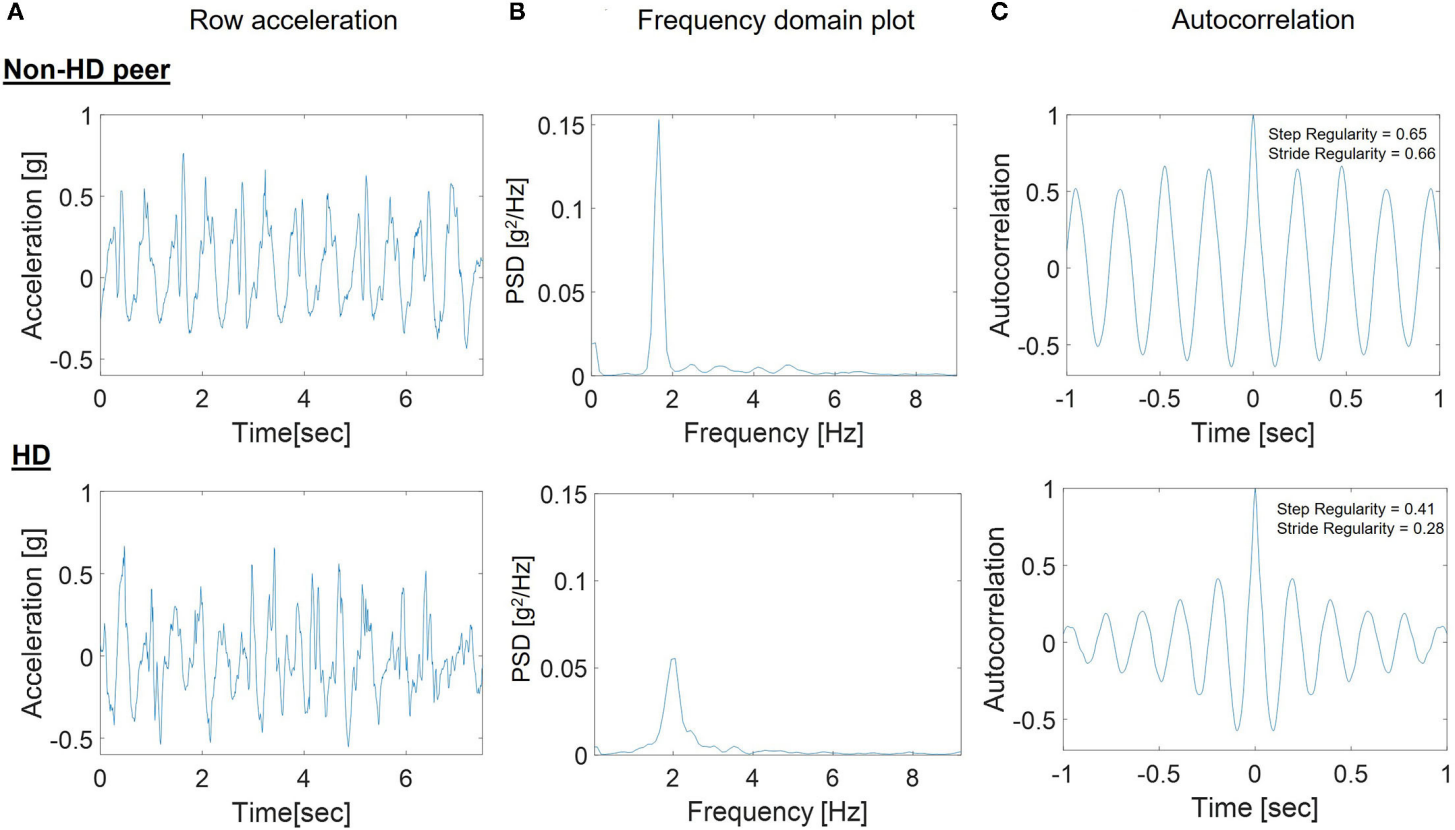
Example steps in the processing of the 7-day accelerometer data for a participant with HD and a non-HD peer. **(A)** Raw SVM acceleration signal in one bout of walking; **(B)** Frequency domain plot used to determine the peak in the frequency domain for a bout of walking; **(C)** Autocorrelation plot used to determine stride regularity in the bout. The peak in the frequency domain **(B)** was much lower in the HD participant [*p*(f) = 0.06] than in the non-HD peer [*p*(f) = 1.67] and the autocorrelation plots **(C)** show that the step and stride regularity was significantly lower in the HD participants compared to non-HD peers.

Figure 2 provides an illustrative example of the accelerometer signal (2A) from a walking bout of one HD participant without irregular movements compared to a non-HD peer, along with the corresponding frequency domain analyses (2B) and autocorrelation plot (2C). These signals are the basis of the gait quality measures. Step regularity and the peak in the frequency domain were lower in the HD participant, indicating a less consistent and less regular walking pattern. The peak in the frequency domain (2B) was much lower in the HD participant (*p*(f) = 0.06) than in the non-HD peer (*p*(f) = 1.67). The autocorrelation plots (2C) show that the step and stride regularity was significantly lower in the HD participants compared to non-HD peers, reflecting a less consistent and more variable gait pattern.

Group differences in daily-living gait quality are summarized in Table 2. Cadence was lower in the HD participants compared to non-HD peers (*p* = 0.04). Within-bout walking consistency, as represented by amplitude, width, step regularity, stride regularity and step time variability were significantly different between HD and non-HD peers (*p* < 0.001 for all comparisons). These differences indicate that the within-bout gait of the people with HD was more variable and less consistent than that of the non-HD peers (similar to the example shown in Figure 2). Group differences in bout-to-bout changes (i.e. variations across the week) in daily-living gait quality are summarized in Table 3. HD participants had lower across-bout variability for all measures except for the SD of the dominant frequency measure, which was similar in both groups (*p* = 0.347) and for the SD of the width of the dominant frequency, which was higher in HD than in non-HD peers (*p* = 0.003). In contrast, to the other measures of consistency, higher width reflects lower consistency, so essentially this latter finding is consistent with the other results, which indicate lower bout-to-bout consistency in the HD participants compared to the non-HD peers.

**Table 3:** Bout-to-bout changes in daily-living gait quality in HD participants and non-HD peers.

		**HD** **participants (*n* = 28)**	**Non-HD** **peers (*n* = 20)**	***P-*value**	**Effect size**
Rhythm	SD of Cadence (step/min)	6.90 (3.27)	9.31 (5.47)	0.002^*^	0.43
	SD of Dominant Frequency (Hz)	0.17 (0.09)	0.16 (0.15)	0.347	0.14
Magnitude	SD of Range (g)	0.42 (0.16)	0.48 (0.49)	0.012^*^	0.36
	SD of RMS (g)	0.05 (0.02)	0.07 (0.12)	0.001^*^	0.46
Consistency /	SD of Amplitude (unitless)	0.15 ± 0.05	0.21 ± 0.03	<0.001^*^	1.52
Regularity	SD of Width (Hz)	0.21 ± 0.09	0.15 ± 0.03	0.003^*^	0.91
	SD of Step Regularity (unitless)	0.13 ± 0.04	0.18 ± 0.03	<0.001^*^	1.14
	SD Stride Regularity (unitless)	0.15 ± 0.04	0.18 ± 0.02	0.002^*^	1.03
	SD of Step time CV (%)	6.70 ± 1.88	9.25 ± 1.49	<0.001^*^	1.56

* *Significant after Bonferroni correction (p = α/components; α= 0.05). Entries are reported as mean+/-SD or median (IQR) determined on each participant across all walking bouts that were equal or larger than 30 sec. SD, Standard deviation*.

**Figure 3:**
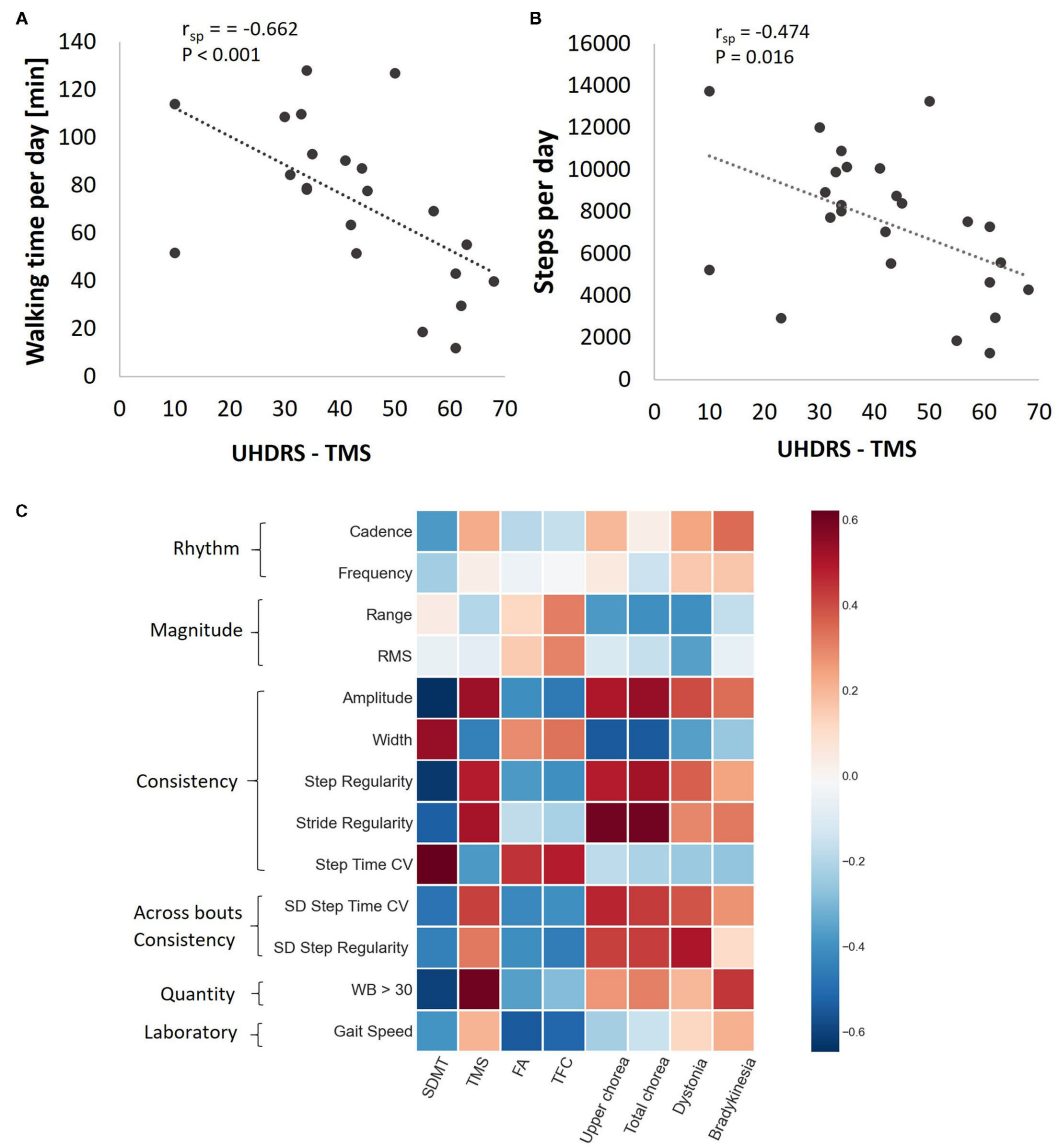
Associations between daily-living measures and clinical measures among the HD participants. Walking time per day **(A)** and steps per day **(B)** as a function of TMS score. r_sp_, Spearman's rank correlation coefficient after adjusting to age and gender. **(C)** Heat map showing the Spearman correlation coefficients between gait quality measures and HD clinical scores, after adjusting to age and gender. Darker pixels reflect higher correlation values. Note that while the rhythm measures tended to be only mildly correlated with the clinical measures, the consistency measures were generally more strongly correlated with the clinical measures.

Relationships between clinical measures and accelerometer-derived daily-living measures are summarized in Figure 3. Among the participants with HD, UHDRS-TMS scores were negatively correlated with two measures that reflect the amount of daily-living walking [walking time per day (Figure 3A) and steps per day (Figure 3B)]. Within-bout and across-bout gait consistency measures (e.g., amplitude, width, step and stride regularity) were moderately (r~0.6) correlated with the clinical measures of upper extremity chorea and total chorea (Figure 3C).

## Discussion

This study reports accelerometry-derived measures of everyday walking in people with manifest HD. Uniquely, we used wrist-worn devices to assess daily-living walking in people with HD in the community while accounting for involuntary, irregular movements. This study considered the potential influence of involuntary movements during walking when using accelerometry-derived measures for remote monitoring in HD. We found that daily-living step counts and time spent walking among a heterogeneous group of HD participants were not different from that of non-HD peers; nonetheless, these measures were inversely correlated with disease severity (UHDRS-TMS). HD participants also tended to have fewer long walking bouts (defined as periods of walking ≥30 sec) compared to non-HD peers, perhaps reflecting fatigability (29) or limited lifespace (30, 31). This is in contrast to recent work in people with dementia, who showed differences only in very short walking bouts (<10 sec) between dementia subtypes (32). Recent work found that continuous gait monitoring can discriminate between people with mild Alzheimer's Disease (AD) and non-AD peers (22). Our findings provide further support for continuous gait monitoring in HD and the importance of context and real-world gait assessment.

In this study, we were able to develop an algorithm, validated by in-person clinical assessments, to identify irregular movements to quantitatively characterize walking behavior in individuals with HD and non-HD peers. Not surprisingly, we found that irregular movements were more common in HD compared to non-HD peers. Interestingly the percentage of irregular movements was almost twice as great in people with relatively high clinician-measured chorea as compared to those with low chorea (recall Figure 1). This finding is similar to that reported by Dinesh et al. (10) in which they examined chorea-like movements in the sitting position. We extend their work by showing, perhaps for the first time, that the amount of walking time spent with irregular movements is significantly higher in people with HD and that this measure is associated with clinical rating of chorea.

Importantly, measures of within-bout and across-bout walking consistency and variability were different in HD participants and non-HD peers, even after considering the irregular, chorea-like movements. This suggests that chorea alone does not account for differences in consistency and variability seen in individuals with HD. Previous research has suggested that parameters of gait variability are a particularly relevant digital biomarker for gait impairment with high sensitivity to differentiating individuals with and without HD mutation (33). Consistent with that, we found that within-bout measures of consistency (in some sense the mirror image of variability) were significantly reduced in HD participants compared to non-HD peers and that these measures were associated with clinical measures of disease severity. The relatively high correlations between the consistency measures and the motor clinical measures (recall Figure 3) suggest that motor symptoms such as chorea and dystonia may influence the ability to maintain a consistent gait. Interestingly, cognitive function, as measured by SDMT, was also related to some of these daily measures. During daily-living, the neurodegenerative processes that limit the ability of individuals with HD to walk with low stride-to-stride variability are clearly manifest, even though other factors (e.g., the environment) may play a role. In the future, it will be interesting to try to tease out and evaluate if these associations reflect cause and effect relationships.

Here, we extend earlier work on stride-to-stride variability in HD (33, 34) and show that across-bout measures of consistency were also different in HD and non-HD peers. Such measures can only be obtained with repeated, “multiple tests”, one of the advantages of daily-living measurement. Adaptability of gait in the community is an essential component of skilled gait and preventing falls (35) and is thus an important metric for evaluating interventions targeting gait and falls. We found reduced across-bout walking variability (as measured by SD of measures of rhythm, magnitude, and consistency/regularity) in participants with HD compared to non-HD peers, suggesting that individuals with HD were less able to adapt their gait in a daily-living environment. This parallels earlier findings in older adults with mild cognitive impairment (20). Future research should evaluate the mechanisms that contribute to this apparent lack of adaptability among HD participants and to examine responsiveness to therapeutic interventions. Moreover, it would be interesting to investigate the grouping of gait variables into several domains by applying factor analysis.

The group differences for several of the gait quality measures (both within and across bouts) had relatively high effect sizes (>1.0). They were higher than the effect sizes for daily-living gait quantity and for those obtained for in-lab gait speed. This suggests that these measures may be more useful as clinical endpoints than the amount of daily-living walking and conventional in-lab measures. Larger effect sizes could potentially allow for smaller sample sizes in clinical studies. In particular, step regularity, which represents the within-bout walking consistency of the step pattern, had a very large effect size. Replicating the effect sizes seen in our data (which were between HD and non-HD peers) longitudinally in HD intervention studies would further support the utility of these metrics as clinical endpoints.

### Limitations

This study has several limitations. The sample included only individuals with early-mid stage HD and did not include individuals with premanifest or prodromal HD. This would be useful to determine if differences in gait quantity and quality can be detected earlier in the disease process. It would also be interesting to examine if the observed changes in daily-living are related to CAG mutation length. We also only evaluated upper extremity movements in relation to their impact on gait. It is possible the irregular movements of the trunk or lower extremity could further impact quality or quantity. However, adding additional sensors on other extremities would increase participant burden and potentially change or limit regular movement in the home environment (i.e., decrease ecological validity of the sensor).

This was also a cross-sectional study with a relatively small number of participants. Initial validity was demonstrated; however, other clinimetric properties of these wrist-worn derived measures still need to be established (e.g., minimal clinically important differences). Future research should evaluate the algorithm in another set of patient data and monitor individuals longitudinally to evaluate the trajectory of changes in gait quality and quantity over time and in response to interventions.

## Summary

This study provides proof of concept demonstrating the utility of metrics representative of daily-living gait quantity and quality in individuals with HD over 7 days while accounting for the influence of irregular movements. This is the first study to evaluate wrist-worn accelerometry to evaluate daily-living physical activity and gait in HD, with ground truth established from clinical measures. Gait impairments are a salient feature of HD, and with the use of wrist-worn devices, we generated measures of gait quality and quantity that may be useful clinical endpoints for a range of clinical interventions. This study also provides useful information about the importance of gait quality as a sensitive measure of motor impairment in HD. Activities that address gait adaptability in daily living, as well as the consistency and regularity of gait patterns, may be important targets of rehabilitation interventions. In summary, the present results suggest that multiple measures of daily-living walking are altered in people with HD and that a subset of these measures is associated with clinician-based tests of chorea and disease severity. These findings set the stage for larger-scale, follow-up studies to further evaluate these possible digital biomarkers and their potential as measures of therapy and disease progression in HD.

## Data Availability Statement

We aim to make our research data available wherever possible, subject to regulatory approvals, any terms and conditions placed upon us from external providers, patient confidentiality and all laws concerning the protection of personal information.

## Ethics Statement

We received Institutional Review Board/Ethics approval at all sites: Cardiff University, Cardiff, UK (study sponsor; School of Healthcare Sciences Research Ethics Committee, approval granted 29 June 2017), Teachers College, Columbia University, New York, NY, USA (IRB 17-322), New York State Psychiatric Institute, New York, NY, USA (IRB 7488), Wayne State University, Detroit, MI, USA (IRB 1701000248), George Huntington Institute, Münster, Germany (2017-079-fs), and Tel Aviv Medical Center, Tel Aviv, Israel (0090-17-TLV). The patients/participants provided their written informed consent to participate in this study.

## Author Contributions

All authors made substantial contributions to the conception or design of the work or the acquisition, analysis or interpretation of data or the creation of new software used in the work, drafted the work or revised it critically for important intellectual content, approved the version to be published, and agree to be accountable for all aspects of the work in ensuring that questions related to the accuracy or integrity of any part of the work are appropriately investigated and resolved.

## Funding

This study was funded by the Jacques and Gloria Gossweiler Foundation and sponsored by Cardiff University. Centre for Trials Research, Cardiff University received infrastructure funding from Health and Care Research Wales and Cancer Research UK.

## Conflict of Interest

The authors declare that the research was conducted in the absence of any commercial or financial relationships that could be construed as a potential conflict of interest.

## Publisher's Note

All claims expressed in this article are solely those of the authors and do not necessarily represent those of their affiliated organizations, or those of the publisher, the editors and the reviewers. Any product that may be evaluated in this article, or claim that may be made by its manufacturer, is not guaranteed or endorsed by the publisher.
