# Supplementary material for: Quantification of Daily-Living Gait Quantity and Quality Using a Wrist-Worn Accelerometer in Huntington's Disease
Source: Front Neurol. 2021 Oct 27;12:719442. doi: 10.3389/fneur.2021.719442 (PMC8579964; doi:10.3389/fneur.2021.719442)
Supplement: Supplementary file 1 [file Data_Sheet_1.pdf]

Quantification of Daily-Living Gait Quantity and Quality using a Wrist-worn Accelerometer

Supplementary Information (SI)

In order to develop a gait detection algorithm that could be applied in the presence of irregular, chorea-like movements, data from 42 participants with HD and 14 non-HD peers were used. The data contained triaxial accelerometer (Opal sensors; 120 Hz sampling rate) signals from both wrists, legs, and trunk. Participants performed a standardized laboratory test-based circuit of functional activities required for independent activities of daily living. This included quiet sitting for 30 seconds, writing a sentence, drinking a 100 ml glass of water, 5-repetition chair stand test, Timed Up & Go (TUG) test, 2-minute walk test, step-ups, and quiet standing with eyes open and closed. These assessments were video recorded (GoPro video recorder, GoPro, San Mateo, CA) to assist recognition of movement features and anomalies during data analysis.

Automatic Identification of Walking Bouts

The data was saved locally on the sensors and transferred to a personal computer for analysis using MATLAB (MathWorks, Natick, MA, USA) software. The gait detection algorithm is outlined in SI Figure 1. For gait detection, the data was low pass filtered using a 4th order Butterworth filter with a cutoff frequency of 15 Hz and, the DC component was removed (1). For wrist movement detection that is not dependent on angle or orientation information, a combination of the acceleration components x, y, z in the vector magnitude, Euclidean norm, was computed (1):

$$SVM = \text{sqrt}\,(\mathbf{acc_x}^2 + \mathbf{acc_y}^2 + \mathbf{acc_z}^2)$$

An empirical activity threshold was set to **0.10** [g]. The signals were divided into windows with a duration of 6 seconds, with 5-second overlap. The window size was chosen based on previous studies on activity recognition (1,2), which assessed that windows approximately 6 seconds are sufficient to capture activity periodicity from a wrist-worn sensor. A second threshold based on a standard deviation of a minimum of 0.10 [g] was applied to each window of the acceleration signal. Although the algorithm first steps are based on an empirical threshold, the in-lab data is not needed to tune or personalize the algorithm. The thresholds were set based on subsets of participants with HD and non-HD peers that completed the laboratory test session. The power spectral density (PSD) was estimated using Welch's method with a Hamming window. The frequency at the maximum power was calculated and examined in each window and autocorrelation analysis was performed to access consistency and similarity in terms of pattern. Based on those, a decision was made on 1 sec middle window, iteratively (see SI Fig 2). Finally, the decision whether a window is a walk or non-walk bout was made based on majority voting with the condition of a minimum of 6 seconds long or more for a bout to be considered as a walking bout. The algorithm was validated by comparing it to previously validated algorithms of walk detection from sensors placed on the legs. The walking bouts extracted from the wrist algorithm were compared to the walking bouts extracted by an algorithm that used leg signals for walk detection. We conducted a second validation process with 7 wrist signals with 2 hours recording each of non-HD peer participants that were annotated by a GoPro camera.

When this algorithm was applied to in-lab data, the algorithm was able to accurately detect gait and distinguish it from non-walking windows (accuracy: 92.42%; sensitivity: 93.14%; F1=95.87%). When the algorithm was applied on the second data set validation annotated with the

GoPro camera, the algorithm was able to detect gait and distinguish it from non-walking windows with (accuracy: 96.13%; sensitivity: 86.38%; F1=83.84). It is interesting to note the higher accuracy on the second data set, which is slightly counter-intuitive since this is a dataset that differs from the original. Nonetheless, the difference was not large and, as expected, the F1 score was lower. The accuracy is an important factor, but the F1 score, i.e., the weighted average of precision and recall, takes both false positives and false negatives into account provide a complementary way of evaluating the success of the algorithm.

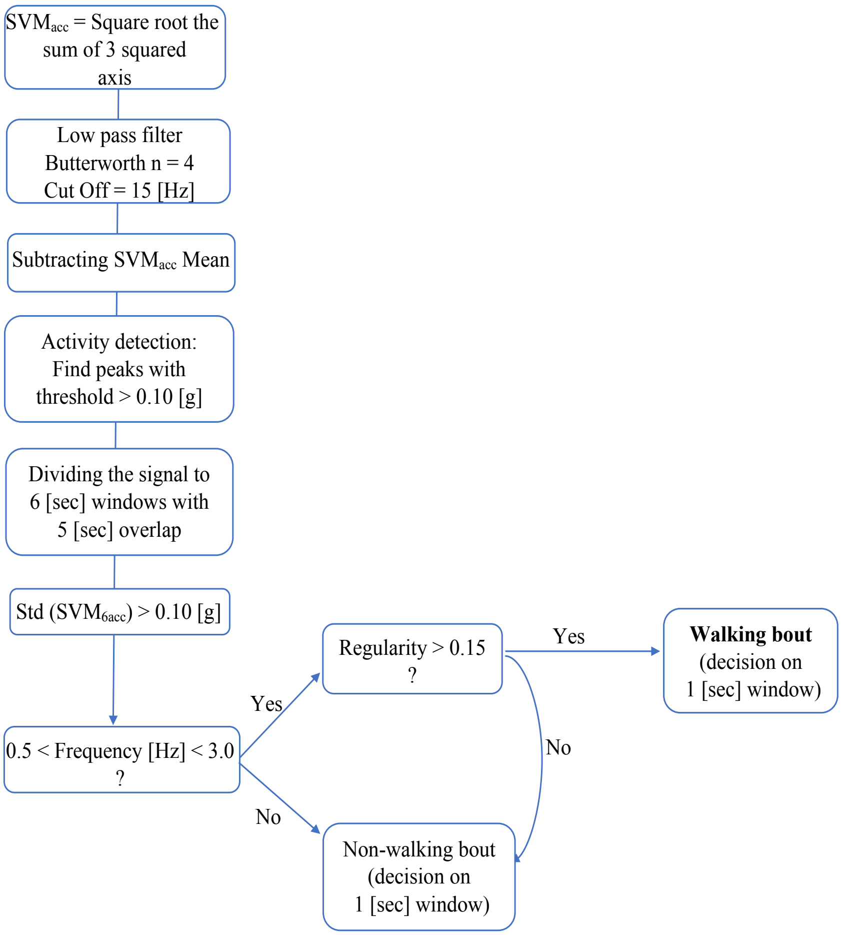

SI Figure 1. A flow chart describing the algorithm detection of walking bouts. SVM_{acc} refer to Euclidean norm of acceleration components. Activity threshold was set empirically**.** Subtraction

SVM_{acc} mean for removing DC component. Frequency refers to the frequency of the dominant peak in power spectra density spectrum. Regularity refers to step and stride regularity values, which are calculated by autocorrelation procedure that estimate the repeating characteristics over a signal sequence containing periodic patterns, while with perfectly periodic patterns the regularity =1.

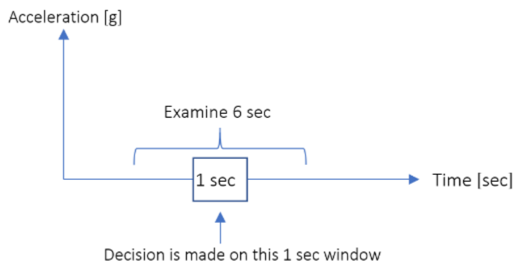

SI Figure 2. A schema presents the process that was made iteratively, **to** determine walk or non-walk on a 1-second long window, based on the signal around it.

Automatic Identification of Walking Bouts with Irregular Movements

The algorithm for irregular walking was developed to detect walking bouts that contain irregular (chorea-like) wrist movements (see SI Fig 2). The detection was based on four feature thresholds extracted empirically from a 2 min walk trial in the lab from eight participants, 3 non-HD peers, and 5 HD participants with different upper body chorea scores. The features: SVM acceleration first and second derivative have been selected with the hypothesis that these features can represent the rapid wrist movement changes and SVM acceleration. Kurtosis and Skewness

features with the hypothesis that irregular movements signals contain more outliers than gait due to higher randomness of the signal. Every walking bout has been divided and checked for the pass of each of the four features within an iteration of 6 seconds window.

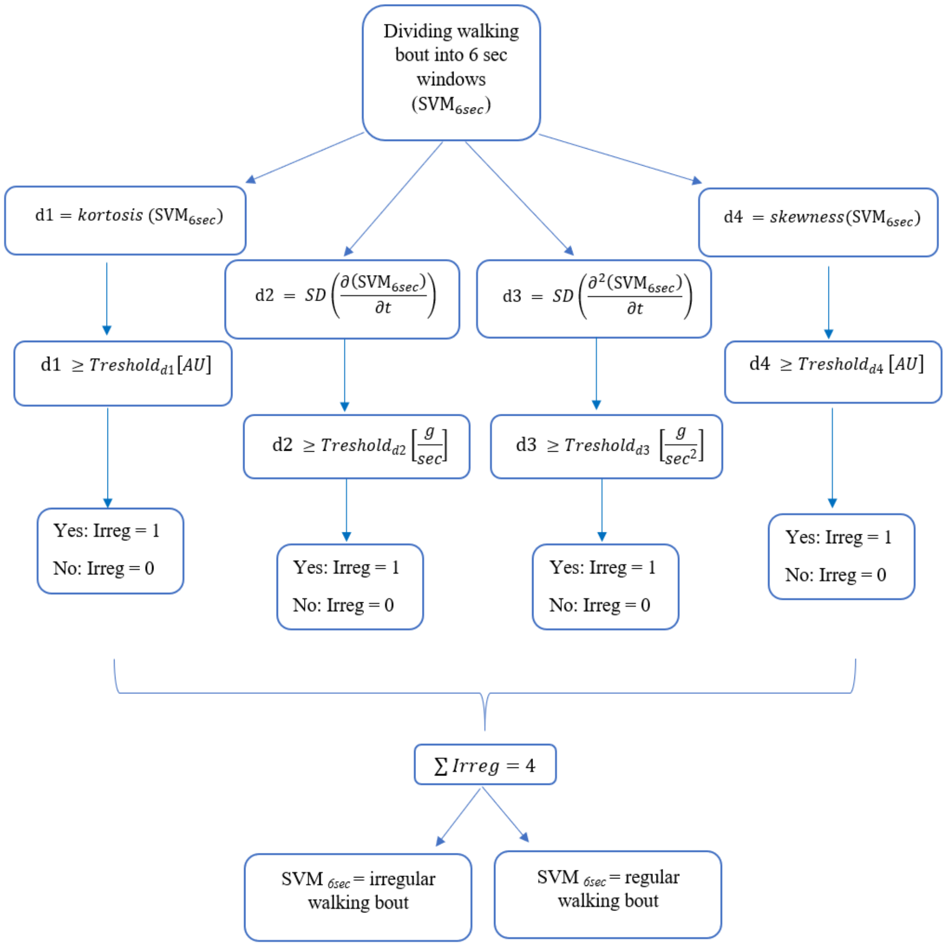

SI Figure 2. A flow chart of: the irregular walking bouts algorithm detection; SD = standard deviation; SVM_{6}sec is Euclidean norm at 6 sec window; Irreg= irregularity score.

Gait measures:

After algorithm validation, walking bouts were extracted and gait analysis performed on 7 days free living wrist signals and laboratory walking test signals from foot and wrist (SI Table 1-3).

Gait quality measures

Rhythm features: The cadence measure was determined by normalizing the average steps to one minute.

Cadence: ((number of WB steps/step time) * 60) (2)

The dominant frequency was determined as the frequency with the largest amplitude in the power spectral density (PSD) spectrum.

Magnitude features: The range measure was defined as the range between the maximum peak value and the minimum peak value of SVM acceleration. The RMS was calculated by the square root of the average squared SVM acceleration.

Variability features: From the dominant frequency, the amplitude peak and the peak width at the half amplitude height were extracted (3,4). The step and stride regularity were calculated using an autocorrelation sequence of the SVM acceleration signal. Autocorrelation is the correlation of a time series with the lagged version of itself; it provides the periodical components embedded in the data. Autocorrelation for the SVM acceleration signal was calculated and normalized to 1.0 at zero lag. The first coefficient peak, next to the zero phase, was attributed to steps as the first dominant period and, the second peak to stride as the second dominant period. The first and second autocorrelation coefficient peak values were defined as the step and stride regularity values, respectively (5).

Step time variability (or coefficient of variation) was defined as the step time standard deviation (SD) divided by the average step time (x 100):

$$\text{Step time variability} = \frac{SD\ (step\ time)}{\text{Mean (step time)}} * 100 \quad (3)$$

Gait quantity measures

For each walking bout that was determined by the walk detection algorithm, the duration was calculated by dividing the length of the walking bout (samples) by the sample rate, 100 samples/sec. The walking time was then converted from seconds to minutes. The walking bout number of steps was calculated by multiplying the walking bout frequency and the duration of the continuous walking bout (6) and the total steps for each day were calculated by summing the steps of all the walking bouts of each day. The total daily-living physical activity was defined by calculating the Euclidean norm of the three axes acceleration measured signal, iteratively over 60 seconds windows during each day. For each day, the mean of all the windows was calculated and the median value of all the days was extracted for each participant (7).

SI Table 1: Daily-living walking quantity in HD and non-HD peers, after removal of irregular movements

	HD Participants	Non-HD Peers	P-value	Effect size
**Walking Time per Day (min)**	59.44 ± 26.80	72.53 ± 16.40	0.059	0.580
**Steps per Day**	6199 ± 2795.56	7854.00 ± 1781.74	0.025	0.696
**Number of Walking Bouts per day**	219.52 ± 73.58	213.52 ± 59.61	0.771	0.089
**Number of Long walking Bouts (≥ 30 seconds)**	19.04 ± 13.23	26.75 ± 8.90	0.032	0.672
**Total daily-living physical activity index (mg)**	42.14 (0.48)	42.71 (0.36)	0.041	0.331

This table summarizes the amount of walking extracted from wrist sensor signals *after* the removal of irregular movements. Entries are presented based on walking bouts equal or longer than 6 seconds; HD participants (n =24) and non-HD peers (n=21). Outcome of condition described in the method section. Values are presented as mean+/-SD or median (IQR).

SI Table 2: In-lab quality measures in HD and non-HD peers based on wrist sensor

		HD Participants	Non-HD Peers	P-value	Effect size
**Rhythm**	Cadence (step/min)	102.17 ± 9.11	102.03 ± 9.13	0.960	0.016
	Dominant Frequency (Hz)	1.73 ± 0.33	1.86 ± 0.26	0.164	0.454
**Magnitude**	Range (g)	1.72 ± 0.45	1.69 ± 0.57	0.827	0.070
	RMS (g)	0.26 ±0.04	0.26 ±0.08	0.748	0.104
**Consistency / Regularity**	Amplitude dominant frequency (unitless)	0.44 (0.24)	0.47 (0.26)	0.721	0.050
	Width dominant frequency (Hz)	1.55 (0.38)	1.66 (0.28)	0.105	0.226
	Step regularity (unitless)	0.43 ± 0.16	0.46 ± 0.14	0.539	0.200
	Stride regularity (unitless)	0.46 ± 0.19	0.56 ± 0.16	0.088	0.557
	Step time variability (%)	27.01(7.89)	31.03(12.7)	0.245	0.161

This table summarizes the gait quality measures extracted from 2 min walk test performed as part of in-lab activity tests from wrist sensor signals. Entries are presented based on walking bouts equal to 30 seconds. HD participants (n=39) and non-HD peers (n=13).

Entries are reported as mean+/-SD or median (IQR).

SI Table 3: In-lab quality measures in HD participants and non-HD peers based on lower limb sensors

		HD Participants	Non-HD Peers	P-value	Effect size
**Rhythm**	Cadence (step/min) [a]	112.12 ± 7.63	115.06 ± 13.41	0.342	0.325
	Dominant Frequency (Hz)	1.86 (0.15)	1.92 (0.16)	0.750	0.046
	Gait speed (m/s)	1.10 (0.28)	1.41 (0.31)	<0.001	0.465
**Magnitude**	Range (g)	4.89 ± 1.27	3.54 ± 1.73	0.011	0.986
	RMS (g)	1.14 ± 0.23	0.95 ± 0.47	0.082	0.661
**Consistency / Regularity**	Amplitude dominant frequency (unitless)	0.79 (0.37)	0.95 (0.21)	0.024	0.333
	Width dominant frequency (Hz)	0.70 (0.04)	0.69 (0.02)	0.299	0.153
	Step regularity (unitless)	0.56 (0.19)	0.48 (0.67)	0.857	0.026
	Stride regularity (unitless)	0.56 ± 0.29	0.54 ± 0.32	0.785	0.102
	Step time variability (%)	-	-	-	-

This table summarizes the gait quality measures extracted from 2 min walk test performed as part of in-lab activity tests from lower limb sensor signals. Entries are presented based on walking bouts of 10 seconds. HD participants (n=38) and non-HD peers (n=9) except as

indicated for cadence; [a] HD (n=42) and non-HD peers (n=11). Entries are reported as mean+/-SD or median (IQR).

References

1. Mannini A, Intille SS, Rosenberger M, Sabatini AM, Haskell W. Activity recognition using a single accelerometer placed at the wrist or ankle. Med Sci Sports Exerc. 2013 Nov;45(11):2193–203.

2. Soltani A, Paraschiv-Ionescu A, Dejnabadi H, Marques-Vidal P, Aminian K. Real-World Gait Bout Detection Using a Wrist Sensor: An Unsupervised Real-Life Validation. IEEE Access. 2020;

3. Herman T, Weiss A, Brozgol M, Giladi N, Hausdorff JM. Gait and balance in Parkinson's disease subtypes: objective measures and classification considerations. J Neurol. 2014 Dec;261(12):2401–10.

4. Weiss A, Brozgol M, Dorfman M, Herman T, Shema S, Giladi N, et al. Does the evaluation of gait quality during daily life provide insight into fall risk? A novel approach using 3-day accelerometer recordings. Neurorehabil Neural Repair. 2013 Oct;27(8):742–52.

5. Moe-Nilssen R, Helbostad JL. Estimation of gait cycle characteristics by trunk accelerometry. J Biomech. 2004 Jan;37(1):121–6.

6. Kang X, Huang B, Qi G. A Novel Walking Detection and Step Counting Algorithm Using Unconstrained Smartphones. Sensors (Basel). 2018 Jan 19;18(1):E297.

7. Galperin I, Hillel I, Del Din S, Bekkers EMJ, Nieuwboer A, Abbruzzese G, et al. Associations between daily-living physical activity and laboratory-based assessments of motor severity in patients with falls and Parkinson's disease. Parkinsonism Relat Disord. 2019 May;62:85–90.
